# Effects of Ionizing Radiation on Physical Properties of Peripherally Inserted Central Catheter

**DOI:** 10.1371/journal.pone.0162837

**Published:** 2016-09-09

**Authors:** Jian Zhang, Shichuan Zhang, Lintao Li, Yan Xing, Maoqiu Cao, Jinhua Wu, Bin Jiang, Ting Zhang

**Affiliations:** 1 Department of Radiation Oncology, Sichuan Cancer Hospital, Chengdu, Sichuan, China; 2 Department of Thoracic Surgery, Sichuan Cancer Hospital, Chengdu, Sichuan, China; Northwestern University Feinberg School of Medicine, UNITED STATES

## Abstract

Peripherally inserted central catheter (PICC) has been widely used to treat cancer patients. It is unknown whether or not it can be applied safely during radiotherapy. The study aimed to investigate the direct effects of gamma radiation on physical properties of PICC. A total of 60 catheters were included in this study. Thirty PICCs were exposed to a radiation field, and another 30 PICCs received radiation in a 3-cm homogeneity water equivalent phantom and then were irradiated. Each group was divided into three subgroups: 10 PICCs were given conventional fractionation, 2 Gy per fraction, 5 fractions per week; 10 PICCs were continuously given hypofractionation, 10 Gy per fraction, for 6 weeks; and 10 PICCs were given mock radiation as controls. The physical properties of these catheters were analyzed after radiation. None of the PICCs leaked under 300-kPa airflow pressure lasting 15 seconds. Fracture force values and liquid velocity values of all PICCs were within the normal range. The liquid velocity values of the control groups were higher than the two groups that received radiation (*P* < 0.05), and there was no difference between the two irradiation groups (*P* > 0.05). There were no statistical differences among the conventional fractionation group, hypofractionation group, and control group when compared to the fracture force values in two parts (*P* > 0.05). The physical property of PICC is quite stable with a clinically relevant dose of gamma radiation. It is likely that PICC can be used safely in patients receiving radiotherapy, although further in vivo and clinical studies are required.

## Introduction

Concurrent chemoradiotherapy is one of the major modalities in cancer treatment [1, 2]. To minimize the toxicity of chemotherapeutics, various intravenous accesses have come about. To choose the most appropriate venous infusion method for intermittent chemotherapy, the nurse specialist should assess the patient's wishes, symptoms, vascular conditions, chemotherapy history, current radiotherapy treatment, and economic conditions so that patients may benefit from reducing repeated peripheral venipunctures [3, 4].

Recently, peripherally inserted central catheters (PICCs) have been widely used because of the multiple advantages they offer, including convenient placement by a nursing team, cost-effectiveness, and safety for long-term access [5, 6]; however, they also come with complications. These complications include some common problems regarding venous access, such as luminal occlusion and malpositioning, and also morbid complications, such as deep vein thrombosis (DVT) and bloodstream infection [7–12]. In addition, patients with central vascular access device (CVAD) need to return to the hospital for dressing changes. The interval between changes is based on the type of dressings [13]. This can be inconvenient for outpatients.

There are a number of studies discussing complications, nursing measurements, and education of PICCs in patients receiving chemoradiotherapy; however, little research explores the direct effect of ionizing radiation on PICC. In fact, the safety of PICC in patients receiving radiotherapy is not well documented. Bai et al. suggested that patients should undergo PICC catheter puncture after radiotherapy to reduce complications [3]. Nevertheless, Chopra et al. rated interval placement of PICCs with each cycle of chemotherapy treatment as the most appropriate strategy [14].

This study’s objective was to explore the effect of conventional ionizing radiation on the physical properties of PICCs and to obtain evidence to support using PICCs during radiotherapy.

## Materials and Methods

### Materials

A total of 60 18G (4-Fr) × 65 cm single lumen PICCs (Becton and Dickinson Company, Utah) and a cobalt-60 teletherapy unit (GWX J80, Nuclear Power Equipment Manufacturing Company, China) were used in this study.

### Study design

The PICCs were exposed to radiation under two different conditions. Thirty PICCs were irradiated directly in a radiation field. Another 30 PICCs were embedded in a 3-cm homogeneity water equivalent phantom and then received radiation. In each condition, PICCs were divided further into three groups according to different fractionated regimens. Ten PICCs were given conventional fractionation in 2 Gy per fraction, 5 times per week, Monday through Friday. Ten PICCs were given hypofractionation in 10 Gy per fraction, continuously and consecutively, once a week, for 6 weeks. As controls, 10 PICCs were not given radiation.

### Physical properties evaluation

After the radiation was completed, these PICCs were sent to a third-party detection institution (Foshan Health Line Medical Products, China) for analysis of their physical properties, including fracture force, liquid velocity, and leakage performance. This analysis was done according to the pharmaceutical industry standard of the People's Republic of China (YY0285.1B、YY0285.3A、YY0285.1C、YY0285.1D).

#### Fracture force

Values were applied for catheters with a gauge length of 20 mm at a speed of 400 mm per minute. The reference value range of fracture force of 4-Fr PICC was above 1.3N.

#### Liquid velocity

Values were applied for catheters under 1-m water column pressure that lasted 60 seconds. The reference value range of liquid velocity of 4-Fr PICC was above 3 ml/min.

#### Leakage performance

The catheters were under 300-kPa airflow pressure that lasted 15 seconds. The catheters without leakage were recognized as having good leakage performance.

### Statistical analysis

Mean and standard deviation were used for statistical description. The difference of intragroup measured data was compared with the analysis of variance; the comparison between every two groups was analyzed with the Student–Newman–Keuls test. Analysis was performed using an SPSS Windows package (version 16.0, SPSS, Inc., Evanston, IL, USA).

## Results

Table 1 presents the results of physical property inspection of a total of 60 PICCs. No leakage was observed in all 60 PICCs under 300-kPa airflow pressure that lasted 15 seconds. Fracture force values and liquid velocity values of all PICCs were within the normal range.

**Table 1:** Physical Properties of 4-Fr BD PICCs (*n* = 60).

Group	Fractionation Regimen	*n*	Leakage Performance (yes/no)	Liquid Velocity (ml/min)	Fracture Force (*N*)	Qualified Rate (%)
**A**	**Conventional**	10	no	3.42±0.31	11.04±1.57	100
	**Hypofractionation**	10	no	3.51±0.32	10.25±1.93	100
	**Control**	10	no	3.90±0.29	10.17±1.36	100
**B**	**Conventional**	10	no	3.57±0.38	9.47±2.06	100
	**Hypofractionation**	10	no	3.52±0.37	9.34±1.82	100
	**Control**	10	no	4.08±0.54	9.65±1.63	100

Group A, PICCs were exposed to gamma radiation directly; Group B, PICCs were irradiated in a 3-cm homogeneity water equivalent phantom.

Interestingly, the liquid velocity values of all irradiated groups were lower than the control groups that received mock radiation (*P* < 0.05) (see Table 2), although none of the values were out of the normal range. There was no difference in liquid velocity between the two irradiation groups (*P* > 0.05).

**Table 2:** Comparison of LVCGRG (n = 60, ml/min).

Group	Fractionation Regimen			F	P-value ^a^
	Conventional	Hypofractionation	Control		
**A**	3.42±0.31	3.51±0.32	3.90±0.29	6.85	0.004
**B**	3.57±0.38	3.52±0.37	4.08±0.54	5.05	0.013

Group A, PICCs were exposed to gamma radiation directly; Group B, PICCs were irradiated in a 3-cm homogeneity water equivalent phantom.

^a^ *p* value were calculated using the analysis of variance.

There were no statistical differences among the conventional fractionation group, hypofractionation group, and control group regarding fracture force values (*P* > 0.05) (see Table 3).

**Table 3:** Comparison of Fracture Force in the Control Group and Radiation Groups (n = 60, N).

Group	Fractionation Regimen			F	P-value ^a^
	Conventional	Hypofractionation	Control		
**A**	11.04±1.57	10.25±1.93	10.17±1.36	0.86	0.43
**B**	9.47±2.06	9.34±1.82	9.65±1.63	0.07	0.93

Group A, PICCs were exposed to gamma radiation directly; Group B, PICCs were irradiated in a 3-cm homogeneity water equivalent phantom.

^a^ *p* value were calculated using the analysis of variance.

## Discussion

There were some restrictions on using PICCs for patients with radiotherapy in China. According to the nursing practice standards for intravenous therapy from the National Health and Family Planning Commission, PICCs should not be placed in body sites receiving radiotherapy. Reasons for this may include that some patients need to be in a particular position for radiotherapy, such as lung cancer patients who need to keep their upper arm elevated during treatment [15]. Also, there were risks of catheter fracture and movement due to inadvertent force during the radiotherapy process [16]. A well-trained nursing team, as well as comprehensive and detailed education, would ensure PICC being used with slight complication [17]; however, because of the little evidence regarding radiation impact on PICC, the Michigan Appropriateness Guide for Intravenous Catheters (MAGIC) suggested that interval placement of PICCs with each chemotherapy treatment may be the most appropriate strategy [14]. Such strategy needs repeated intravenous punctures that increase a patient’s cost and pain and may result in higher complication rates [18].

In the current study, the catheters were first exposed directly to radiation in radiation fields. The results showed that there is a difference in liquid velocity between the irradiated group and control groups. In the second experimental setting, PICCs were embedded in a homogeneity water equivalent phantom, which is a commonly used tissue substitute. The embedded depth of 3 cm was determined by taking into account the percentage of depth dose (PDD) curve for 60 Co gamma beams and the depth of PICC placed in a human body. Again, the results showed that there was a small but significant difference in the fluid velocity values of PICCs between the irradiation and no irradiation groups. In principle, the velocity was related to the inner radii of PICCs. Regardless of spurious effect, the slight difference might due to radiation-induced cross-linking reaction. All physical property values were within the reference ranges. In addition, neither conventional fractionated radiation therapy nor hypofractionated radiation caused catheter leakage in the experiments.

The current study provided preliminary results showing that ionizing radiation, either in small or large fraction sizes, has little effect on the physical property of PICC. Therefore, the concern that radiation may impair PICC quality is not supported by this study.

The finding that the liquid velocity was relatively small in irradiated catheters is very interesting. Currently, the underlying mechanism is not known. The difference may be caused by the change in molecular level of the catheters or simply due to the small sample size. Studies with a larger number of catheters and spectral analysis on catheters are thus warranted.

Another limitation of the current study is that it is an in vitro study. Compared with the experimental condition, catheters in patients’ bodies are under more complicated situations. Some biological factors, such as blood pressure and coagulation of platelets and phagocytes, may affect catheters along with radiation. Furthermore, a 60 Cobalt teletherapy unit with an average of 1.25 MeV energy of gamma ray was used in the current study. In clinics, most patients received higher energy X-rays, such as 6 MV or 8MV; therefore, in vivo animal experiments are planned to confirm the in vitro findings. In addtion to photons, other radiation modalities, such as proton and heavy charged particles, may also be included in future studies. Some patients received PICC-based concurrent chemoradiotherapy. The catheters from these patients will be gathered to analyze the high-energy X-ray radiation effect on their physical properties.

## Conclusions

PICCs are widely used on cancer patients; however, current safety information of radiation on catheter use is absent. This leads to a concern of PICC application during radiotherapy. This study showed that when catheters were targeted and irradiated either in conventional or large fraction sizes using gamma rays, their physical properties were quite stable. The liquid velocity had a very small change in irradiated catheters, but the values were still within the normal range. To our knowledge, this study is the first study investigating the possible impact of radiation on PICCs. The results provided the first evidence that ionizing radiation is likely safe for PICC, although further in vivo and patient-based studies are needed.

## Supporting Information

S1 File Raw Data of 60 PICCs. This includes raw data relating to the results of physical property inspection of 60 PICCs.
(XLS)
